# Landscaping tuberculosis multimorbidity: findings from a cross-sectional study in India

**DOI:** 10.1186/s12889-024-17828-z

**Published:** 2024-02-13

**Authors:** A. Chauhan, M. Parmar, J. D. Rajesham, S. Shukla, K. C. Sahoo, S. Chauhan, S. Chitiboyina, A. Sinha, G. Srigana, M. Gorla, Sanghamitra Pati

**Affiliations:** 1 Public Health Foundation of India, New Delhi, India; 2 World Health Organization, Country Office for India, New Delhi, India; 3 State Tuberculosis Cell, Hyderabad, Telangana India; 4 World Health Organization, National Tuberculosis Elimination Programme - Technical Support Network, New Delhi, India; 5 Indian Council of Medical Research-Regional Medical Research Centre, Bhubaneswar, Odisha India; 6 State TB Training and Demonstration Centre, Hyderabad, Telangana India

**Keywords:** Prevalence, Tuberculosis, Multimorbidity, Chronic conditions

## Abstract

**Background:**

Multimorbidity, the concurrent presence of two or more chronic conditions is an emerging public health challenge. Till date, most of the research have focused on the presence and interaction of selected co-morbidities in tuberculosis (TB). There exist a critical knowledge gap on the magnitude of multimorbidity among TB patients and its impact on health outcomes.

**Methods:**

We undertook a cross-sectional study to assess the prevalence and patterns of multimorbidity among newly diagnosed TB patients in two states of India. A total of 323 patients were interviewed using a structured multimorbidity assessment questionnaire for primary care (MAQ-PC). MAQ-PC is already validated for Indian population and elicits 22 chronic conditions. We defined TB multimorbidity as the co-existence of TB with one or more chronic conditions and identified commonly occurring dyads (TB + single condition) and triads (TB + two conditions).

**Results:**

More than half (52%) of TB patients reported multimorbidity. Among dyads, depression, diabetes mellitus (DM), acid peptic disease (APD), hypertension, chronic alcoholism, arthritis and chronic back ache (CBA) were the most common co-occurring conditions while ‘DM + arthritis’, ‘depression + APD’, ‘depression + DM’ were the most commonly occurring triads among TB patients. Factors such as increasing age, low levels of education, alcohol abusers, drug-resistant TB and having health insurance were significantly associated with multimorbidity among TB patients.

**Conclusions:**

Our findings suggest high prevalence of multimorbidity among newly diagnosed TB patients in India. The presence of concordant and discordant conditions with TB may increase the health complexity, thus necessitating appropriate care protocols. Given, the current situation, wherein TB and non-communicable diseases (NCD) services are delivered through collaborative framework between programmes, there is a need for addressing multimorbidity at the healthcare delivery level.

## Introduction

Global tuberculosis (TB) report, 2022 reported an estimated 10.6 million people developed TB and 1.4 million people died from TB worldwide in 2021 [1]. India, the highest TB burden country in the world, contributed to more than one fourth (28%) of the global TB incident cases in 2021 [1]. India is committed to end TB by 2025, i.e. achieve the Sustainable Development Goals 2030 targets for TB, five years ahead [2]. However, its efforts are impeded by the rising prevalence of non-communicable diseases (NCDs), communicable disease (CDs) such as COVID-19, HIV infection, viral hepatitis, etc. and other determinants of health such as undernutrition [3]. Improvements in the living conditions, changing lifestyle and progress in healthcare effectiveness has resulted in an increase in chronic conditions [4]. Especially, multimorbidity, the presence of two or more chronic conditions has become a norm rather than exception [5]. It is different from co-morbidity, which refers to the combined effects of additional conditions in relation to the index condition in an individual [5]. 

Multimorbidity in TB (TB multimorbidity), defined as co-existence of TB with one or more chronic condition is an emerging public health issue in India [3]. TB frequently co-occurs with NCDs such as mellitus (DM), depression and cancer considered to be risk factors for TB [3]. TB leads to breakdown of immune system leading to increase in one’s susceptibility to other chronic conditions whereas chronic CDs such as HIV, long Covid-19, viral hepatitis adversely interact with TB at cellular and molecular level [6–8]. Thus, a bidirectional interplay exists between TB and other chronic conditions. Moreover, presence of multimorbidity in TB complicates the outcomes and vice versa [7]. Thus, TB multimorbidity is more complex to manage than concordant multimorbidity (where conditions have shared pathophysiology or shared approaches to management) [5]. TB multimorbidity is a peculiar challenge for India owing to a rising burden of NCDs, in addition to widespread prevalence of HIV and other infectious diseases.

TB multimorbidity may represent a substantial burden for individuals, increasing their risk of adverse events and unfavorable outcomes ultimately affecting the already constrained healthcare system [9]. It poses a primary concern for healthcare system in India which is typically focused to manage single conditions [10]. Much insight has been gained in the recent years in terms of our understanding of TB co-existing with single chronic conditions such as HIV or DM but evidence on the burden, characteristics, and clusters of TB multimorbidity is lacking. Further, such estimates may better inform health services managers for interwoven management of multimorbidity into TB care. With this background, we aim to assess the burden, socio-demographic co-relates and patterns of TB multimorbidity in India.

## Methodology

### Study design and participants

**Fig. 1:**
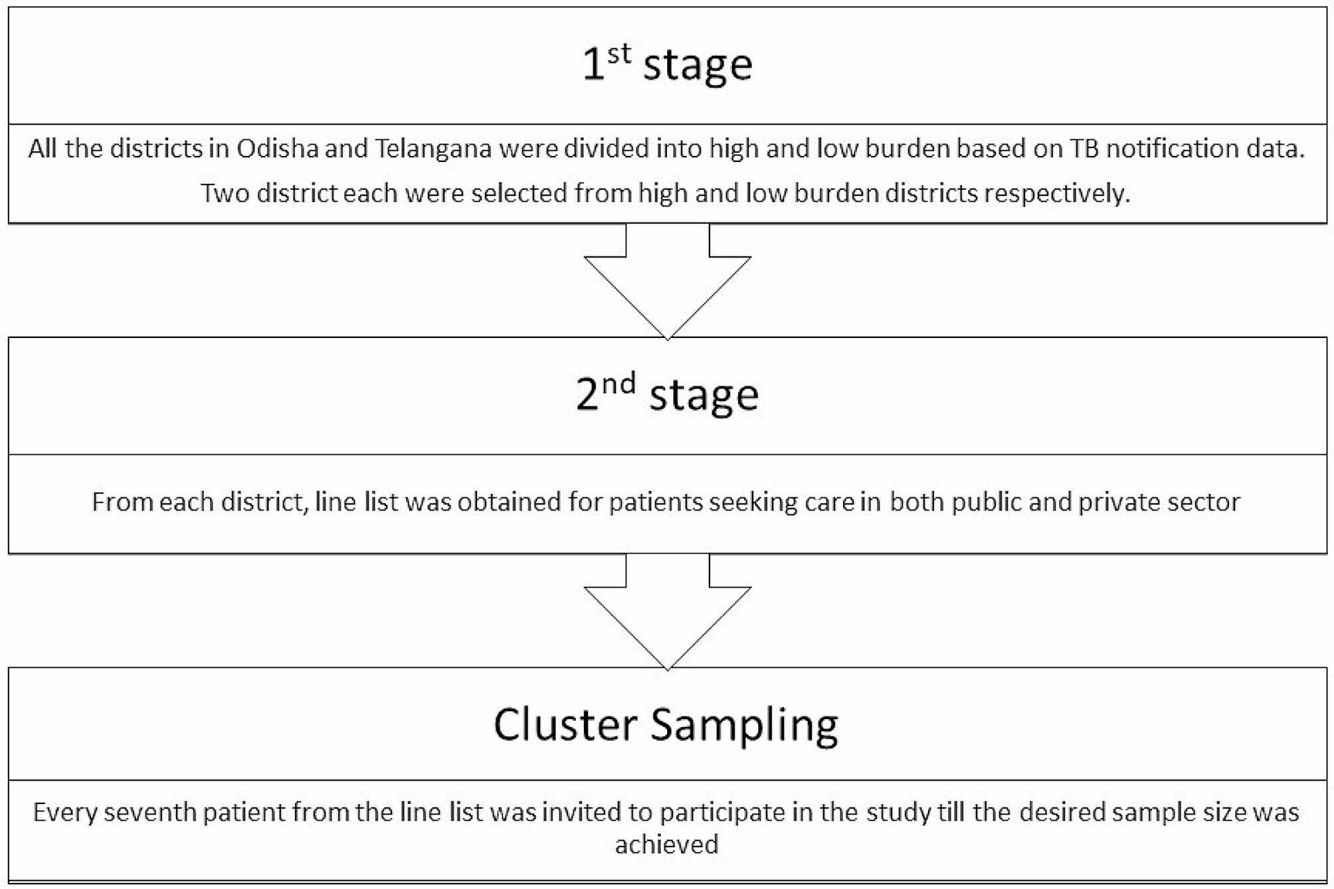
Two-stage stratified cluster sampling

We undertook a cross-sectional study from October 2022 to March 2023 in Telangana and Odisha, India. Odisha is a province located in the eastern coast and Telangana located in the southern part of India has health and socio-demographic indicators similar to the national average [11, 12]. As the district TB centers (DTC) are the cornerstone of the National TB Elimination Program (NTEP) for organizing and providing preventive, promotive and curative TB care to patients [13], we decided to conduct our study at the DTCs. We adopted a two-stage cluster stratified random sampling method for recruiting the health facilities. In first stage, all districts of the two states (30 in Odisha and 33 in Telangana) were divided into two clusters i.e., high burden and low burden as per the TB notification data from NTEP, Government of India [14]. From each cluster, two DTCs were randomly selected in each state. Each of the four DTC had the line list of TB patients registered in the previous six months. This included patients seeking care in both the public as well as private sector. From the line list, every seventh patient was randomly selected and interviewed virtually (Fig. 1). Virtual telephonic interviews of TB patients were conducted using Multimorbidity Assessment Questionnaire - primary care (MAQ-PC) tool to identify multimorbidity [15]. For patients whose contact details were not available or incorrect or those who do not wish to participate, the next available TB patient was contacted.

Sample size: In absence of availability of data on prevalence of TB multimorbidity in India, we estimated the sample size based on the prevalence of DM among TB reported by Jarde et al. as 22% [3]. It was estimated as 304 with *p* < 0.05 level of significance, 95% confidence interval, design effect of 1.05 and 10% drop-out rate.

### Inclusion and exclusion criteria

We included patients aged 18 years or above attending the facilities with confirmed diagnosis of TB irrespective of the drug susceptibility status, who provided the consent. Patients too ill to participate, those with insufficient cognitive ability to complete the questionnaire, those with debilitating physical and mental disabilities and those not willing to participate were excluded from the study.

### Data collection

To collect data, we used a pre-developed and validated structured tool– MAQ-PC. This was translated into the vernacular language (Oriya and Telugu) and validated for its use among TB patients. The detailed methodology for development and validation of our tool is available elsewhere [15]. The multimorbidity subscale explored the presence of any of the 21 listed self-reported chronic diseases. Open options for “any other conditions” were added to capture unlisted conditions if any. We followed the prescribed guidelines for analysis of Physical Health Questionnaire-9 (PHQ-9) towards diagnosing depression [16]. 

To avoid duplication, unique identification numbers were given to the patients and who have already been interviewed in any of the facility. Interviews were conducted by four well trained field investigators with a nursing background well versed with local language and patient history taking. Each interview spanned from 20 to 30 min.

### Ethical approval

The study adhered to the Declaration of Helsinki principles and was approved by the Institutional Ethics Committee of ICMR-RMRC, Bhubaneswar (Vide no. TRC-IEC-173/13). Prior permissions were obtained from the respective state and district TB officers. At the offset, information about the study was communicated with each participant and an explanation of the study objectives and procedures were given. They were provided sufficient time to understand and interview schedule was fixed after due diligence and consultation as per their convenience. Any queries were resolved prior to the interview and understanding was facilitated. Informed verbal consent was obtained prior to the interview. All the participants were granted the right to withdraw from the study at any time and to withhold the information if required. Necessary steps were taken to preserve patient anonymity and confidentiality.

### Data analysis

All the data were analyzed by SPSS 27.0 version. Descriptive statistics were calculated and presented as proportion, mean, and standard deviation (SD). Data was compared using Pearson’s chi-square test and independent sample t test for categorical and continuous variables respectively. Difference between sub-groups was measured by Odd’s ratio with 95% confidence intervals. A p-value of < 0.05 was taken as statistically significant. Patterns (Dyads and Triads) were described using a simple descriptive statistical method employing exhaustive analysis of all possible combination of two or three co-morbid conditions using [17]. Dyads were the combination of TB with other single chronic condition and triad were the combination of TB with two other chronic conditions.

## Results

A total of 323 eligible patients with TB were interviewed, consisting of 61.9% males and a mean age of 43.87 ± 16.64 years. Approximately 52.9% were young adults aged 18–45 years, 55.9% were residing in rural area and 65.5% were covered by health insurance. More than one-third of the participants had no formal education and 65.6% were belonging to lower socio-economic status. Majority were drug susceptible TB patients (94.4%) and 85.3% had pulmonary TB.

**Fig. 2:**
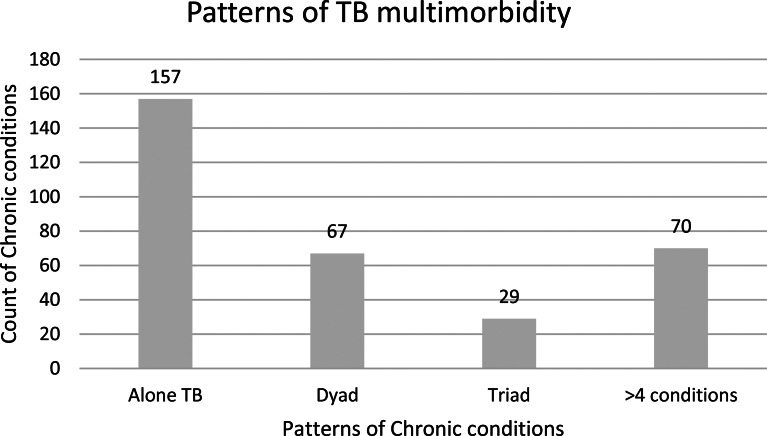
Patterns of TB multimorbidity

TB multimorbidity (TB with one or more chronic conditions) was observed in 52.01% of the TB patients (Fig. 2) while 30.6% had more than two and 21.67% had four or more chronic conditions. The average number of chronic conditions among TB patients was 1.34 (SD, 1.87).

More than half of the TB patients without multimorbidity were young adults, males, had drug susceptible pulmonary TB and belonging to low socio-economic status. While a similar profile was observed for the participants having TB with multimorbidity except majority were more than 45 years old, 41.1% were females, 54.41% resided in urban/semi-urban areas, 71.32% were from low socio-economic status, 10% had drug resistant TB patients, 29.23% were alcohol abusers 20% were tobacco abusers and 83.09% had health insurance (Table 1).

**Table 1:** Socio-demographic profile of the study participants

Attributes (N = 323)	Categories	Total TB patients n (%)	TB patients with Multimorbidity Absen n (%)	TB patients with multimorbidity present n (%)
Age (years)	18–30	97 (30.03)	69 (36.90)	28 (20.59)
	31–45	74 (22.91)	37 (19.79)	37 (27.21)
	46–60	94 (29.10)	49 (26.20)	45 (33.09)
	≥ 61	58 (17.96)	32 (17.11)	26 (19.12)
Gender	Female	123 (38.08)	67 (35.83)	56 (41.18)
	Male	200 (61.92)	120 (64.17)	80 (58.82)
Residence	Rural	180 (55.90)	118 (63.44)	62 (45.59)
	Urban	77 (23.91)	37 (19.89)	40 (29.41)
	Semi-urban/Slum	65 (20.19)	31 (16.67)	34 (25.00)
Caste	General	78 (24.15)	38 (20.32)	40 (29.41)
	Other Backward Class	150 (46.44)	95 (50.80)	55 (40.44)
	Scheduled Tribe	18 (5.57)	4 (2.14)	14 (10.29)
	Scheduled Caste	77 (23.84)	50 (26.74)	27 (19.85)
Education	No formal education	135 (41.80)	83 (44.39)	52 (38.24)
	Primary	42 (13.00)	27 (14.44)	15 (11.03)
	Secondary	82 (25.39)	44 (23.53)	38 (27.94)
	Higher	64 (19.81)	33 (17.65)	31 (22.79)
Occupation	Currently working	77 (23.84)	31 (16.58)	46 (33.82)
	Homemaker	83 (25.70)	34 (18.18)	49 (36.03)
	Currently not working	11 (3.41)	1 (0.53)	10 (7.35)
	Never worked	152 (47.06)	121 (64.71)	31 (22.79)
Economic Status	Low	212 (65.63)	115 (61.50)	97 (71.32)
	Middle	103 (31.89)	69 (36.90)	34 (25.00)
	High	8 (2.48)	3 (1.60)	5 (3.68)
Alcohol abuser	No	207 (82.14)	115 (94.26)	92 (70.77)
	Yes	45 (17.86)	7 (5.74)	38 (29.23)
Tobacco abuser (*N* = 252)	No	221 (87.70)	117 (95.90)	104 (80.00)
	Yes	31 (12.30)	5 (4.10)	26 (20.00)
Site of tuberculosis	Extra-pulmonary	37 (14.68)	12 (9.84)	25 (19.23)
	Pulmonary	215 (85.32)	110 (90.16)	105 (80.77)
Drug susceptibility status	Drug resistant	14 (5.56)	1 (0.82)	13 (10.00)
	Drug susceptible	238 (94.44)	121 (99.18)	117 (90.00)
Health Insurance available	No	111 (34.47)	88 (47.31)	98 (52.69)
	Yes	211 (65.53)	23 (16.91)	113 (83.09)

Depression (33.1%) was the most commonly occurring chronic condition, followed by DM (14.5%), acid peptic disease (APD) (14.2%), hypertension (11.4%), and chronic alcoholism (11.4%) whereas ‘DM + arthritis’ (26.7%), ‘depression + APD’ (20.8%), ‘depression + DM’ (17.2%), ‘DM + hypertension’ (16.6%) and ‘depression + CBA’ (16.6%) were the most commonly occurring triad among TB patients. (Figs. 1 and 2; Table 2).

**Table 2:** Prevalence of chronic conditions among TB patients (Dyads and Triads)

Condition	n (%)
**Dyads with TB**	
Depression	107 (33.1)
DM	47 (14.5)
APD	46 (14.2)
Hypertension, Chronic Alcoholism	37 (11.4), 37 (11.4%)
Arthritis	31 (9.5)
CBA	31 (9.5)
Chronic Heart Disease	23 (7.12)
Dementia	17 (5.28)
Visual Impairment	16 (4.97)
Thyroid Disease	13 (4.04)
Hearing Impairment	11 (3.42)
Chronic Lung Disease	9 (2.7)
Stroke	4 (1.25)
HIV	3 (0.9)
Chronic Kidney Disease	3 (0.93)
Epilepsy	2 (0.62)
**Triads with TB**	
DM + Arthritis	45 (26.7)
Depression + APD	35 (20.8)
Depression + DM	29 (17.2)
DM + Hypertension	28 (16.6)
Depression + CBA	28 (16.6)
Depression + Hypertension	25 (14.9)
DM + APD	23 (13.6)
Depression + Arthritis	22(13.09)
Arthritis + APD	20 (11.9)
Depression + Chronic Alcoholism	20 (11.9)
Depression + Chronic Heart Disease	17 (10.1)
Hypertension + APD	16 (9.5)
DM + CBA	15 (8.9)
Arthritis + Hypertension	12(7.1)
Arthritis + CBA	12 (7.1)
Depression + Thyroid	10 (5.95)
Hypertension + CBA	9 (5.3)
DM + Chronic Heart Disease^1^	6 (3.5)
Arthritis + Chronic Heart Disease	5 (2.9)

*Cancer, Filariasis, Other Mental Disorders, Kala Azar/Leprosy, Hepatitis, Long COVID-19 were not prevalent in our study population

Table 3. shows the association between socio-demographic characteristics and multimorbidity. Factors such as increasing age, low levels of education, alcohol abusers, drug-resistant TB and having health insurance were significantly associated with multimorbidity among TB patients.

**Table 3:** Association between TB multimorbidity and various socio-demographic attributes

Attributes	Categories	Adjusted Odds Ratio (95% CI)
Age (years)	18–30	Reference
	31–45	**6.76 (2.17–21.02)**
	46–60	**10.46 (3.11–35.20)**
	≥ 61	**19.55 (4.33–88.34)**
Gender	Female	Reference
	Male	1.62 (0.46–5.68)
Residence	Rural	Reference
	Urban	1.64 (0.59–4.52)
	Semi-urban/Slum	2.70 (0.99–7.35)
Caste	General	1.21 (0.47–3.12)
	Other Backward Class	Reference
	Scheduled Tribe	4.44 (0.71–27.65)
	Scheduled Caste	2.41 (0.85–6.89)
Education	No formal education	**0.25 (0.07–0.91)**
	Primary	**0.16 (0.04–0.66)**
	Secondary	**0.33 (0.11–0.99)**
	Higher	Reference
Occupation	Currently working	2.21 (0.75–6.49)
	Homemaker	4.38 (1.22–15.67)
	Currently not working	22.66 (1.75-292.77)
	Never worked	Reference
Economic Status	Low	Reference
	Middle	0.46 (0.18–1.17)
	High	1.63 (0.13–20.96)
Alcohol abuser	No	Reference
	Yes	**10.42 (3.04–35.68)**
Tobacco abuser	No	Reference
	Yes	3.23 (0.83–12.58)
Site of tuberculosis	Pulmonary	Reference
	Extra-pulmonary	4.25 (1.36–13.25)
Drug susceptibility status	Drug suceptible	Reference
	Drug resistant	**143.85 (7.31-2832.13)**
Health Insurance	No	Reference
	Yes	**4.71 (1.70-13.04)**

TB patients with multimorbidity reported a fair to poor self-rated health compared to those without multimorbidity and this was statistically significant. (*p* < 0.0001) (Table 4).

**Table 4:** Self-rated health among TB patients with and without multimorbidity

Self-rated Health	Multimorbidity Absent n, %	Multimorbidity Present n, %	Chi-square Test
Very Good	64 (52.46)	20 (15.38)	< 0.00001
Good	44 (36.07)	59 (45.38)	
Fair	10 (8.20)	45 (34.62)	
Poor	4 (3.28)	6 (4.62)	

*Significant at *p* < 0.05

## Discussion

This study observed multimorbidity to be common among TB patients. Especially, it was prevalent among older males from low socio-economic status residing in urban areas. Depression, DM, APD, hypertension, chornic alcoholism, arthritis and CBA were the most common dyad observed with TB whereas DM and arthritis, depression and APD, depression and DM, DM and hypertension, depression and CBA were the most commonly occurring triad among TB patients. Factors such as increasing age, low levels of education, alcohol abusers, drug-resistant TB and having health insurance were significantly associated with multimorbidity among TB patients.

Our study found a 52% prevalence of multimorbidity and 30.6% prevalence of more than two chronic conditions among TB patients. Similar to our findings, Chen et al. in their study in China observed 38.6% and 21.2% whereas Karl et al. in South Africa reported 26.9% and 25.3% prevalence of multimorbidity and two or more chronic conditions respectively among TB patients [9, 18]. This suggest multimorbidity to be a norm rather than exception among TB patients, especially among the high TB burden countries in the world. TB is a chronic inflammatory disease, which itself is a risk factor for other chronic conditions [7]. Contrastingly, Barbara et al. in Brazil, based on the analysis of national TB notification database (SINAN) reported a prevalence of 1.1% for more than two chronic conditions [19]. One of the reasons for such low prevalence of multimorbidity was cited as optional reporting of chronic conditions in the SINAN database. Similarly, Giri et al. in their analysis of data from Ni-kshay database reported 7.6% prevalence of DM with TB and 0.08% prevalence of DM and HIV with TB in Odisha, India [20]. The national TB notification database, Ni-kshay has been streamlined to capture various information related to TB in India [14]. Except HIV, DM and tobacco abuse, other chronic conditions are not routinely screened and recorded resulting in a lower reported prevalence of TB multimorbidity except in DR-TB patients [21]. Thus, our findings emphasize the need for capturing full spectrum of multimorbidity through a structured assessment tool. This will help in designing tailored interventions and improve patient outcomes.

We found TB with or without multimorbidity to be common among those with low literacy and low socio-economic status. Historically in India, TB has been denoted as a condition of poverty and low socio-economic status [22]. Health inequalities have a strong association between lower levels of education and poorer health outcomes [23]. And health literacy, the degree to which individuals have the capacity to obtain, process, and understand basic health information to make appropriate health decisions is the mechanism through which the level of education affects health outcomes among TB patients [24]. Our own systematic review found limited levels of health literacy among TB patients, especially those with TB and DM (Unpublished). Thus, improving health literacy needs to be contemplated as an essential mechanism to improve outcomes by the NTEP in India by addressing multimorbidity.

India is targeting to end TB by 2025 i.e. achieving the SDG 2030 milestones, five years ahead [2]. To achieve this, it is essential to address TB multimorbidity. We found depression, DM, APD, hypertension, arthritis and CBA as the most commonly clustering conditions. A meta-review conducted by Jarde et al. also found a 45% prevalence of depression, and 17.7% prevalence of DM among TB patients [3]. An analysis of World Health Survey in 48 LMICs found a strong association between TB and depression (OR ≥ 3) [25]. TB is a stigmatized condition, associated with discrimination and lack of social support [26]. It has a chronic paradigm with long-duration of treatment [13]. This itself affects the mental health of the TB patients. Depression affects medical adherence and nutritional status which are important attributes of successful TB treatment outcomes and recovery [3]. Thus, TB and depression mutually impact each other [3, 13]. Currently, under the Ayushman Bharat Yojana, Ayshman Arogya Mandir (AAM) in India, there is a provision of psychologist and counsellors whose services can be utilized to address this issue [27]. Similarly, DM is also a major challenge among TB patients owing to a synergistic interplay [3]. We found a 14.0% prevalence of DM. However, owing to the nature of the study design, chronology could not be determined and thus it is difficult to interpret which condition occurred first. Also, DM among TB patients in the initial stages is associated with a transient hyperglycemic stage [28]. Hence, future studies must ascertain the level of multimorbidity among newly diagnosed TB patients and follow longitudinally to determine the temporality and trajectory. Such understanding would enable policy makers to develop suitable interventions and avert adverse outcomes.

Our study observed APD and arthritis to be common among TB patients. Similar findings were observed by Pati et al. in their study in India [17]. At the same time, both APD and arthralgia are the commonly reported side-effect of anti-tuberculosis treatment as well as 10–11% of extra-pulmonary TB manifest as TB bone and joint disease [29, 30]. In order to delineate whether this is a co-existing chronic condition or associated with anti-TB treatment, follow up studies among newly diagnosed TB patients are required. Clinicians treating TB should also recognize commonly occurring conditions which may impact the quality of life of patients. We found a commonly occurring triad of hypertension, TB and arthritis. This poses a challenge for treating physician for prescribing treatment [7]. Primary prevention for hypertension involves weight reduction and increased physical activity [31]. However, this cannot be recommended to those with joint problems. This suggests complexity in management of such patients [3]. The existing TB comorbidity collaborative framework and guidelines may consider for such complexities for a shared responsibility to manage multimorbidity in India.

### Strengths and limitations

Our work revealed the multimorbidity burden and patterns among TB patients using a structured, validated and an exhaustive tool to assess TB multimorbidity in India for the first time. This indicates the complexity associated with management of discordant (conditions unrelated to each other) multimorbidity. However, we restricted interviewing the patients to healthcare facility and the magnitude may be different in the community settings. Also, the TB multimorbidity was ascertained through self-reporting, which itself is associated with recall bias. Nonetheless, the validity and reliability of self-report is well established in our previous study. Further, due to the design of the study, the causality and temporality of TB multimorbidity could not be determined. To better understand the trajectory of TB multimorbidity, more longitudinal studies among newly diagnosed TB patients involving follow-ups are needed. Despite these limitations, our study provides a much-needed understanding of clustering and burden of multimorbidity among TB patients which may paved way to designing of appropriate solutions.

## Conclusion

Multimorbidity is common among TB patients in India. Current data capturing system must consider incorporation of structed multimorbidity assessment tool such as MAQ-PC to identify other chronic conditions among TB patients. TB multimorbidity might accentuate the existing health inequity and thus, equity dimension needs to be considered while planning for ending TB through multi-disease elimination approaches. Effective joined-up approaches to prevention, screening and treatment of co-occurring conditions are required. National programmes of CDs, NCDs (NPNCD) and TB need to confabulate with each other for inclusion of the assessment tools and management strategy. Findings from our study have significant implications for program managers and policy makers, particularly for comprehensive and coordinated management of multimorbidity (horizontal amalgamation of NTEP, NPNCD and National Mental Health Program at AAMs) as well as contemplating the need for national TB multimorbidity guidelines and multi-disease elimination initiatives in India as part of the overall health system ambit to achive SDG targets.

## Data Availability

The datasets used and/or analysed during the current study available from the corresponding author on reasonable request.
